# The influence of head-of-bed elevation in patients with obstructive sleep apnea

**DOI:** 10.1007/s11325-017-1524-3

**Published:** 2017-06-24

**Authors:** Fábio José Fabrício de Barros Souza, Pedro Rodrigues Genta, Albino José de Souza Filho, Andrew Wellman, Geraldo Lorenzi-Filho

**Affiliations:** 1 Pulmonary Division - Hospital São José, Pulmonar Sleep Laboratory and Universidade do Extremo Sul Catarinense, Antônio de Lucca 91, 4th floor, Pio Correa, Criciúma, Santa Catarina Brazil; 2 Sleep Laboratory, Pulmonary Division, Heart Institute (InCor), University of São Paulo Medical School, São Paulo, Brazil; 3 Pulmonary Division, Hospital São José and Pulmonar Sleep Laboratory, Criciúma, Santa Catarina Brazil; 4 Division of Sleep Medicine, Brigham and Women’s Hospital and Harvard Medical School, Boston, MA USA

**Keywords:** Obstructive sleep apnea, Therapy, Patient positioning, Polysomnography

## Abstract

**Purpose:**

The purpose of this study is to test the effects of a mild degree of head-of-bed elevation (HOBE) (7.5°) on obstructive sleep apnea (OSA) severity and sleep quality.

**Methods:**

OSA patients were recruited from a single sleep clinic (Criciúma, Santa Catarina, Brazil). Following a baseline polysomnography (PSG), all patients underwent a PSG with HOBE (within 2 weeks). In addition, a subset of patients performed a third PSG without HOBE.

**Results:**

Fifty-two patients were included in the study (age 53.2 ± 9.1 years; BMI 29.6 ± 4.8 kg/m^2^, neck circumference 38.9 ± 3.8 cm, and Epworth Sleepiness Scale 15 ± 7). Compared to baseline, HOBE significantly decreased the apnea-hypopnea index (AHI) from 15.7 [11.3–22.5] to 10.7 [6.6–16.5] events/h; *p* < 0.001 and increased minimum oxygen saturation from 83.5 [77.5–87] to 87 [81–90]%; *p* = 0.003. The sleep architecture at baseline and HOBE were similar. However, sleep efficiency increased slightly but significantly with HOBE (87.2 [76.7–90.7] vs 88.8 [81.6–93.3]; *p* = 0.005). The AHI obtained at the third PSG without HOBE (*n* = 7) returned to baseline values.

**Conclusions:**

Mild HOBE significantly improves OSA severity without interfering in sleep architecture and therefore is a simple alternative treatment to ameliorate OSA.

## Introduction

Obstructive sleep apnea (OSA) is a common disorder characterized by repetitive partial or complete obstruction of the upper airway during sleep [1–3]. The pathophysiology of OSA is complex and is caused by the interplay of both anatomical and non-anatomical factors including neuromuscular responsiveness, ventilatory instability, and arousal threshold [3]. Continuous positive airway pressure (CPAP) is the most common treatment for OSA. However, CPAP adherence is not ideal and may be even worse among subjects with milder forms of OSA [4, 5].

Alternative options for OSA treatment include oral appliances, upper airway surgery, oropharyngeal exercises, and positional therapy [6–12]. Head-of-bed elevation (HOBE) has been shown to effectively reduce OSA severity in small studies [13, 14]. However, HOBE was aggressive and ranged from 30° to 60°, making this approach difficult to be tolerated in clinical practice [13–16]. The evidence that HOBE may be useful to treat OSA also comes from physiological studies showing that HOBE decreases upper airway collapsibility and increases upper airway area as compared to supine position [13–16]. Mild HOBE is widely accepted to treat gastroesophageal reflux [17]. Therefore, mild HOBE may be an attractive and low-cost option to treat OSA either alone or in combination with other therapies. However, there is very little evidence to support HOBE effectiveness in clinical practice. We hypothesized that a mild HOBE (7.5°) will significantly improve OSA without interfering on sleep architecture and efficiency. In order to test these hypotheses, we studied patients with OSA by full polysomnography (PSG) without (baseline) and with HOBE.

## Methods

### Subjects

Men and women aged between 18 and 75 years were recruited from a sleep clinic in Criciúma, Santa Catarina, Brazil. Subjects with a body mass index (BMI) > 40 kg/m^**2**^, a previous diagnosis of heart failure, renal failure, and uncontrolled respiratory or neurological disease, and a baseline PSG showing an apnea-hypopnea index <5 events/h were excluded. The study was approved by the Hospital São José Ethics Committee, Criciúma, Santa Catarina, Brazil (protocol 167/2010). Written informed consent was obtained from all patients before any study procedure. The trial was registered at clinicaltrials.gov (NCT02088723).

### Polysomnography

Polysomnographic recordings were performed using an Alice 5® polysomnography system (Philips Respironics) and comprised of electroencephalogram derivations (F4/M1, C4/M1, O2/M1, F3/M2, C3/M2, O1/M2), electrooculogram, submental and anterior tibialis electromyogram, and electrocardiogram. Airflow was monitored using an oronasal thermistor and a nasal pressure cannula. Thoracic and abdominal movements were registered using inductive plethysmography belts. Body position and oxygen saturation oximetry were also recorded. The studies were manually scored according to the latest American Academy of Sleep Medicine scoring manual [18]. Apnea was defined as a reduction of ≥90% of the oronasal thermistor flow amplitude for ≥10 s. Hypopnea was defined as the reduction ≥30% of nasal pressure flow amplitude for ≥10 s in association with either ≥3% arterial oxygen desaturation or an arousal [18]. Mild, moderate, and severe OSA was classified according to standard criteria (5–14.9, 15–30, >30 events/h, respectively) [19].

### Study design

All patients were evaluated by a clinical interview and Epworth Sleepiness Scale (ESS) questionnaire was applied. A baseline diagnostic polysomnography was performed followed by a polysomnography with HOBE within 2 weeks. The laboratory bed was 2.10 m long and the mattress was at 0.6 m from the floor. A 15-cm-high piece of wood under the head-of-bed legs was used, resulting in a tilt of 7.5°. Because this was not a randomized trial, a third polysomnography without HOBE was performed within the subsequent 2 weeks in a subset of patients in order to exclude that the possible differences in OSA severity between baseline and intervention were not due to random variability.

### Statistical analysis

Statistical analysis was performed using SPSS version 20.0. G Power version 3.0.10 was used for power calculations and *α*˂0.05 was considered significant. The Shapiro-Wilk test was used to test for normal distribution. Paired *t* test or Wilcoxon signed-rank test was used to compare baseline and HOBE data as appropriate. Sample size was calculated expecting a 30% decrease in the AHI after HOBE. A beta of 0.85 and alpha of 0.05 were used and 52 patients were necessary. A repeated measurement ANOVA was performed to test for the difference between the three polysomnographies in the subset of patients that underwent a third PSG without bed elevation. A post-hoc analysis (Tukey’s) was done to test for differences between polysomnographies. A positive response was defined as ≥50% reduction in AHI or AHI < 5 events/h during intervention. A partial response was defined as a decrease in AHI between 25 and 50%. A negative response was defined as <25% AHI reduction.

## Results

**Fig. 1:**
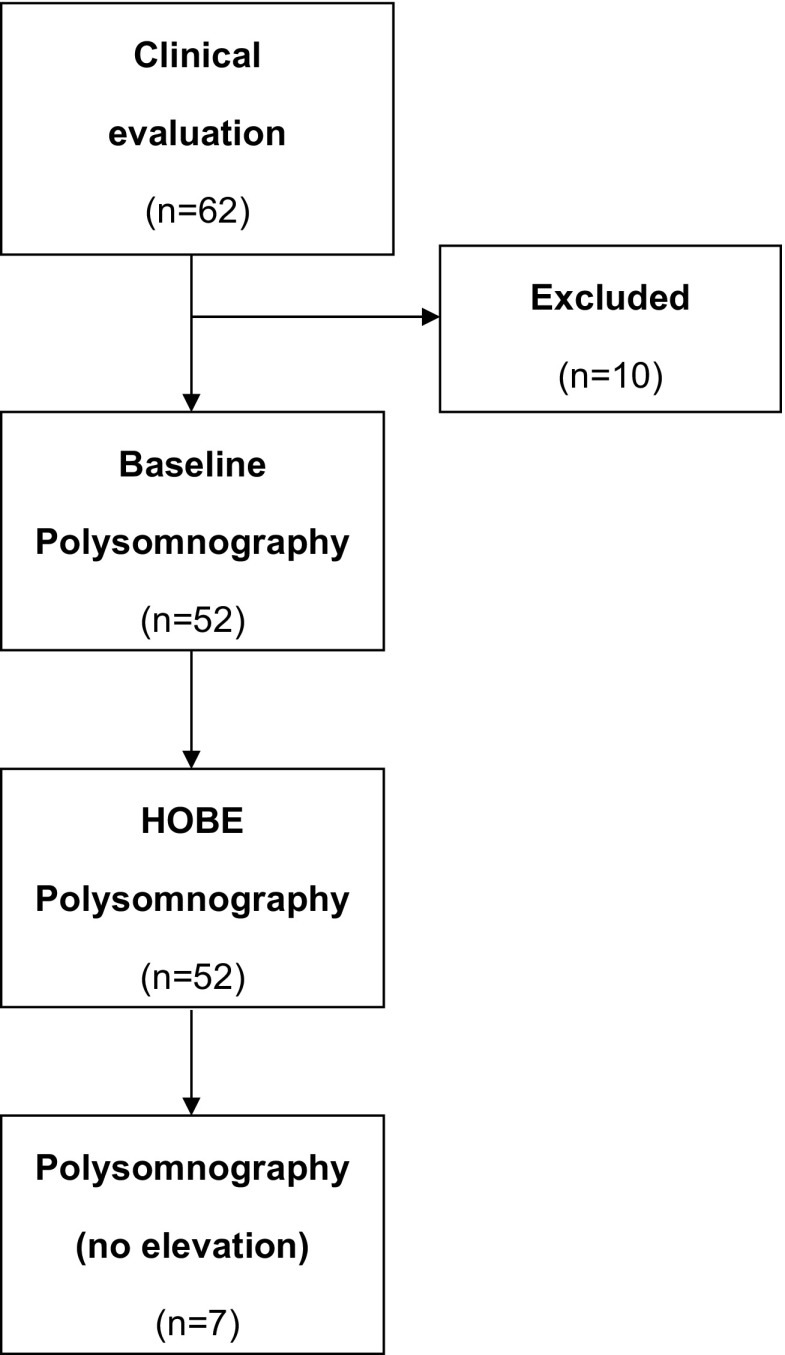
Study flow chart and number of studied and excluded subjects

Sixty-two patients were initially evaluated. Ten patients were excluded because of concomitant chronic obstructive pulmonary disease (*n* = 6), BMI > 40 kg/m^2^ (*n* = 2) and two patients did not agree to participate. Therefore, 52 patients were included in the study. The time lag between the baseline and HOBE PSG was 8.9 ± 2.1 days. A subset of seven patients performed a third PSG without HOBE (9.7 ± 2.6 days after the HOBE PSG) (Fig. 1). The sample consisted of middle aged (53.2 ± 9.1 years) men and women (25 women) that were on average overweight (BMI 29.6 ± 4.8 kg/m^2^, neck circumference 38.9 ± 3.8 cm) with symptoms of excessive daytime sleepiness (EES 14.7 ± 6.9). Patients had, on average, moderate OSA at baseline PSG with an AHI ranging from 5.3 to 44.8 events/h. Twenty, 28, and 4 patients presented mild, moderate, and severe OSA, respectively. The median AHI in baseline PSG was 10.6 [7.6–12.1] events/h in mild, 17.6 [15.7–23.3] events/h in moderate, and 39.4 [36.8–42.4] events/h in severe OSA patients. Table 1 shows the sleep characteristics during baseline and HOBE PSG. HOBE did not promote any significant differences in sleep stages, arousal index (ArI), total sleep time (TST), or time in supine position. Although HOBE was associated with an increase in sleep efficiency (from 87.2% [76.7–90.7] to 88.8% [81.6–93.3], *p* = 0.005), the mean increase was 83.4 ± 9.2 to 91.4 ± 31.6, and this does not appear to be clinically relevant.

**Table 1:** Comparative data between baseline polysomnography and HOBE polysomnography

	Baseline	Head-of-bed elevation	*p*
TST (min)	359 ± 50	365 ± 34	0.446
Sleep efficiency (%)	87.2 [76.7–90.7]	88.8 [81.6–93.3]	**0.005**
N1 (%)	6.0 ± 3.2	5.8 ± 2.5	0.678
N2 (%)	61.9 ± 12.5	60.2 ± 10.9	0.198
N3 (%)	20.7 ± 12.2	21.2 ± 10.1	0.680
REM (%)	11.3 ± 5.6	12.8 ± 6.4	**0.076**
ArI (events/h)	9.1 [5.5–17.7]	8.1 [6.4–12.4]	0.682
Time in supine position (min)	97.8 ± 98.9	107.1 ± 95.6	0.488
AHI (events/h)	15.7 [11.3–22.5]	10.7 [6.6–16.5]	**<0.001**
AHI supine (events/h)	20.5 [10.0–33.2]	11.3 [8.5–22.6]	**0.023**
AHI non-supine (events/h)	12.3 [6.1–27.2]	8.1 [4.4–12.4]	**<0.001**
CAI (events/h)	0 [0–0.3]	0 [0–0.2]	0.321
OAI (events/h)	2.2 [0.9–4.7]	2.1 [0.6–4.4]	0.461
MAI (events/h)	0.2 [0–0.6]	0.2 [0–0.5]	0.257
HI (events/h)	12.9 [8.6–15.4]	6.8 [3.7–11.3]	**<0.001**
SatO_2_ min (%)	83.5 [77.5–87]	87 [81–90]	**0.003**

Results are shown as median [IQR] or mean ± SD

All the *p* < 0.05 in bold

*AHI* apnea-hypopnea index, *CAI* central apnea index, *OAI* obstructive apnea index, *MAI* mixed apnea index, *HI* hypopnea index, *SatO*_*2*_ *min* minimum oxygen saturation, *TST* total sleep time, *N1* non-REM sleep stage 1, *N2* non-REM sleep stage 2, *N3* non-REM sleep stage 3, *REM* rapid eye movement, *ArI* arousal index

**Fig. 2:**
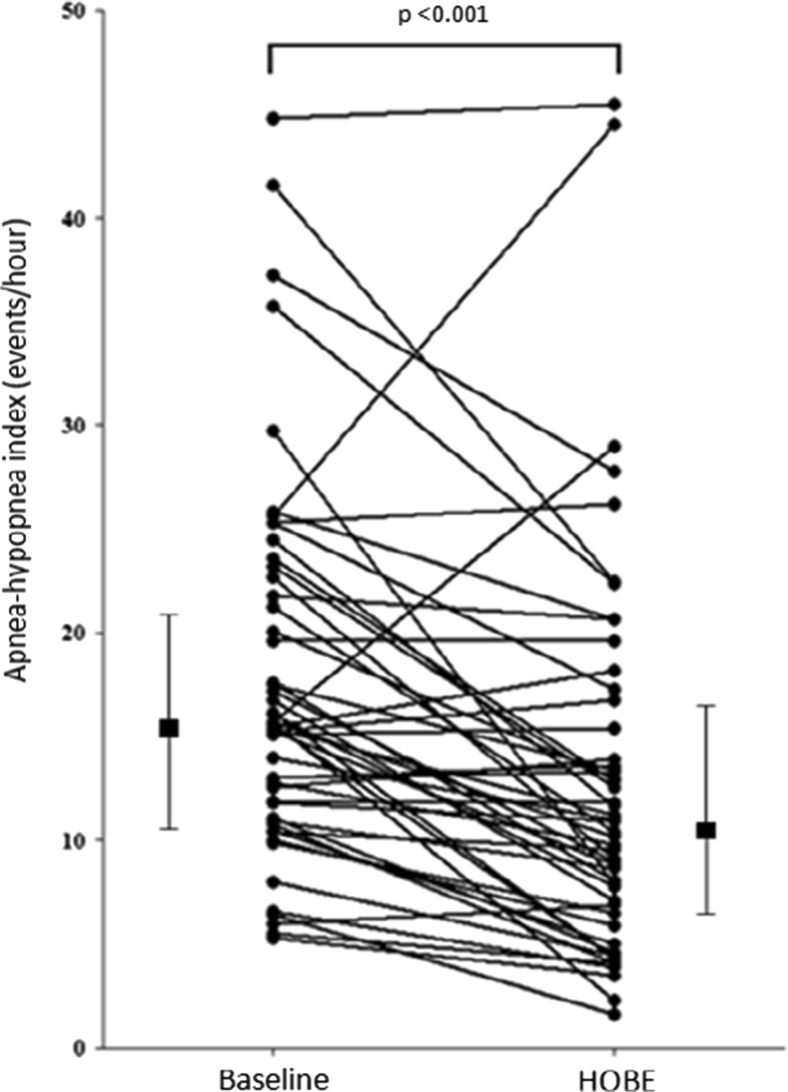
Individual AHI values obtained during baseline and HOBE polysomnographies. *Filled squares* are median values [interquartile range]

There was on average a 31.8% reduction in AHI during HOBE. HOBE significantly decreased AHI from 15.7 [11.3–22.5] to 10.7 [6.6–16.5] events/h (*p* < 0.001). HOBE promoted a greater reduction in the supine AHI (from 20.5 [10.0–33.2] to 11.3 [5.7–34.5] events/h, *p* = 0.023).The individual values of AHI at baseline and HOBE PSG can be observed in Fig. 2. Minimum oxygen saturation increased significantly during HOBE from 83.5 [77.5–87] to 87 [81–90]% (*p* = 0.003). HOBE was most effective to reduce hypopneas (from 12.9 [8.6–15.4] to 6.8 [3.7–11.3] events/h, *p* = 0.001) (Table 1).

Thirteen patients out of 52 patients (25%) presented positional OSA (defined as an AHI at lateral position less than 50% of the AHI at supine position). Among the 13 patients showing positional OSA during baseline PSG, 9 maintained positional OSA with HOBE. Apnea-hypopnea index of the 13 positional OSA patients at the baseline polysomnography (11.9 [10.4–14.3] events/h) did not reduce significantly during HOBE (9.0 [4.6–9.9] events/h), *p* = 0.174.

Thirty-two patients (61.5%) were classified as responders to HOBE (9 patients presented a positive response and 23 patients presented a partial response). In contrast, 20 patients (38.4%) did not respond to HOBE. Among the 7 subjects that underwent a third polysomnography without HOBE, the AHI during the third (22.0 ± 13.6 events/h) and baseline (20.8 ± 10.7events/h) PSGs was similar (*p* = 0.304) but was significantly higher than during the HOBE night (14.0 ± 9.1 events/h) *p* = 0.005.

## Discussion

The major finding of the current study was that a mild degree of HOBE (7.5°) was able to reduce OSA severity by 31.8% on average among patients with predominantly mild to moderate OSA. HOBE had the largest impact in reducing hypopneas (47.3%) and supine-dependent respiratory events (44.9%). We believed that HOBE would reduce sleep efficiency because of some postural discomfort or falling sensation, but this did not occur. HOBE was associated with an increase in sleep efficiency, although this does not appear to be clinically relevant. HOBE may be a simple alternative treatment option for patients with mild to moderate OSA.

The findings of the present study are in line with previous reported small case series. For instance, McEvoy et al. studied 13 male patients during the same night and reported a reduction from 49 ± 5 to 20 ± 7 events/h from supine to sitting position at 60° [13]. Although the authors reported a greater decrease in AHI than in our study, the clinical applicability is limited because patients usually do not tolerate sleeping in the sitting position for prolonged periods. Skinner et al. reported a 22% reduction in AHI in 14 patients with a wide range of OSA severity when sleeping with a shoulder-head elevation pillow [20]. Our study extends these previous findings in a larger sample with clinical applicability by showing a 31.8% reduction in median AHI among patients with predominantly mild to moderate OSA using a HOBE of 7.5°. HOBE was obtained with a 15-cm wedge (5.9 in.). Similar elevation has been shown to be well tolerated and used in studies on gastroesophageal reflux disease [17]. The magnitude of reduction in AHI observed in the present study with HOBE (32%) is similar to that of other non-CPAP alternatives previously reported such as postural position therapy (46%) [21], oropharyngeal exercises (39%) [8]**,** and compression stockings (36%) [22].

The pathophysiology of OSA is complex, may vary from patient to patient, and involves anatomical and non-anatomical components, such as respiratory control stability. The predominant control of hypopneas observed in our study is consistent with other alternative non-CPAP treatments for OSA [23–25]. Similar effects have been observed in therapeutic approaches that act on the respiratory control stability such as acetazolamide [23], dead space, and supplemental oxygen [24] as well as on the mechanical component of OSA such as the instillation of surfactant [25]. Two independent studies reported a significant reduction in pharyngeal critical closing pressure (Pcrit) with HOBE, indicating a protective effect by acting on the anatomical component of OSA. Neill AM et al. showed a significant reduction in Pcrit between natural sleep in the supine position and during 30° HOBE (0.3 ± 2.4 to −4.0 ± 3.2 cmH_2_O, respectively) [14]. In a second study, nine patients studied under general anesthesia with propofol and vecuronium, Pcrit also decreased from the supine to the sitting position at 62° 2.2 (0.84–6.12) to −3.5 (−8.51– −1.32) cmH_2_O, respectively [16]. In line with the physiological research, one study showed a significant increase in the mean upper airway volume (evaluated by cervical computed tomography) from the supine to the head elevation position with 44° (40.35 ± 16.43 vs 48.31 ± 16.21 cm^**3**^, respectively) [26]. HOBE may alleviate the force of gravity on the pharyngeal structures, especially the tongue. The tongue defines the anterior pharyngeal wall which is particularly susceptible to narrowing while sleeping supine [14, 16, 26, 27]. HOBE may also stabilize the upper airway by preventing rostral fluid shift during sleep, a mechanism that may contribute to upper airway obstruction not only in patients with edematous states, but also in typical OSA patients [28].

HOBE is very easy to be applied and well tolerated. HOBE could be a simple measure for patients that are waiting for OSA therapy, such as mandibular advanced device or surgery. HOBE could also be used in combination with other interventions such as oral appliance, oropharyngeal exercises, and weight loss in future studies. HOBE has also the potential of providing a better compliance than other alternative therapies for OSA such as oropharyngeal exercises and positional therapy. For instance, avoiding supine position with tennis ball therapy showed that the long-term patient compliance is poor, with less than 10% of adherence [29].

Our study has several potential limitations. Firstly, this was not a randomized study, and therefore, it is not possible to exclude the impact of difficult to control variables such as the first night PSG effect. However, in a small subgroup of patients that performed a third PSG with no HOBE, it clearly showed a return of OSA severity to the baseline levels. Secondly, one may argue that the effect of HOBE on OSA severity was mild and that a higher degree of bed elevation could be more effective in reducing OSA severity. The elevation adopted in our study was a compromise between effectiveness and comfort. A pilot study (data not shown) showed that patients did not tolerate sleeping with a 20-cm wedge because of sensation of falling. We therefore adopted a bed angulation that is well described in patients with gastroesophageal reflux. Thirdly, the majority of our patients had only mild to moderate OSA, and the effects were more pronounced in reducing hypopneas than apneas. Therefore, the effects of HOBE are more likely to be less evident in patients with severe OSA. Fourthly, HOBE may be contra indicated in patients with specific conditions such as lower limb edema and venous insufficiency.

## Conclusions

HOBE is a simple measure that significantly improves OSA severity without compromising the sleep architecture. This positional intervention reduces AHI and has better effects in hypopnea index and supine AHI. Due its simplicity and low cost, HOBE can be an attractive alternative treatment for OSA.
